# Production of extracellular L-arginase by *Alcaligenes aquatilis* BC2 isolated from soda lakes (Lake Chitu) of Ethiopia

**DOI:** 10.1093/jimb/kuaf017

**Published:** 2025-06-27

**Authors:** Birhan Getie Assega, Kefyalew Ayalew Getahun, Tamene Milkessa, Tsehayneh Geremew Yohannes, Feleke Moges, Mulugeta Aemero, Berhanu Andualem

**Affiliations:** Department of Environmental and Industrial Biotechnology, Institute of Biotechnology, University of Gondar, Gondar, Ethiopia; Department of Pharmacology, School of Pharmacy, College of Medicine & Health Sciences, University of Gondar, Gondar, Ethiopia; Department of Environmental and Industrial Biotechnology, Institute of Biotechnology, University of Gondar, Gondar, Ethiopia; Department of Environmental and Industrial Biotechnology, Institute of Biotechnology, University of Gondar, Gondar, Ethiopia; Department of Medical Microbiology, SBLS, CMHS, University of Gondar, Gondar, Ethiopia; Department of Medical Parasitology, SBLS, CMHS, University of Gondar, Gondar, Ethiopia; Department of Environmental and Industrial Biotechnology, Institute of Biotechnology, University of Gondar, Gondar, Ethiopia

**Keywords:** *Alcaligenes aquatilis* BC2, L-Arginase, Optimization, 16S rRNA, Soda lake

## Abstract

L-Arginase is a therapeutic enzyme that hydrolyzes L-arginine to ornithine and urea. The L-arginase extracted from bacteria has an anticancer activity by causing starvation of nutrients for cancer cells. This study aimed to screen and characterize L-arginase-producing bacteria and to optimize different factors influencing L-arginase production. Isolation and primary screening were carried out by using mineral arginine agar media using phenol red as an indicator. Molecular identification of the isolates was employed by using 16S ribosomal RNA sequencing and phylogenetic tree construction. L-Arginase assay by colorimetric method was carried out to measure the amount of urea liberated from the hydrolysis of L-arginine for quantitative screening. From 31 water samples, 102 colonies were isolated, and those colonies that convert the media to pink were selected as arginase-producing bacteria. 7 isolates were screened from qualitative screening method. Based on quantitative screening, the highest L-arginase was produced from bacteria *Alcaligenes aquatilis* BC2 (92.46 ± 0.19 U/ml) followed by *Paenalcaligenes suwonensis* BCW8 (59.29 ± 0.66 U/ml). Following their mean difference, isolate BC2 was selected for further optimization process of 8 parameters. After optimization, the isolate shows the maximum (163.85 U/ml) enzyme activity. The result of this study implies that novel bacteria were isolated from soda lakes that produce a considerable amount of L-arginase, which can be used as a promising anticancer activity.

**One-Sentence Summary**: This study successfully isolated and characterized a novel L-arginase–producing bacterium, *Alcaligenes aquatilis* BC2, from Ethiopian soda lakes and optimized its enzyme production parameters for potential anticancer applications.

## Graphical Abstract

**Graphical Abstract:**
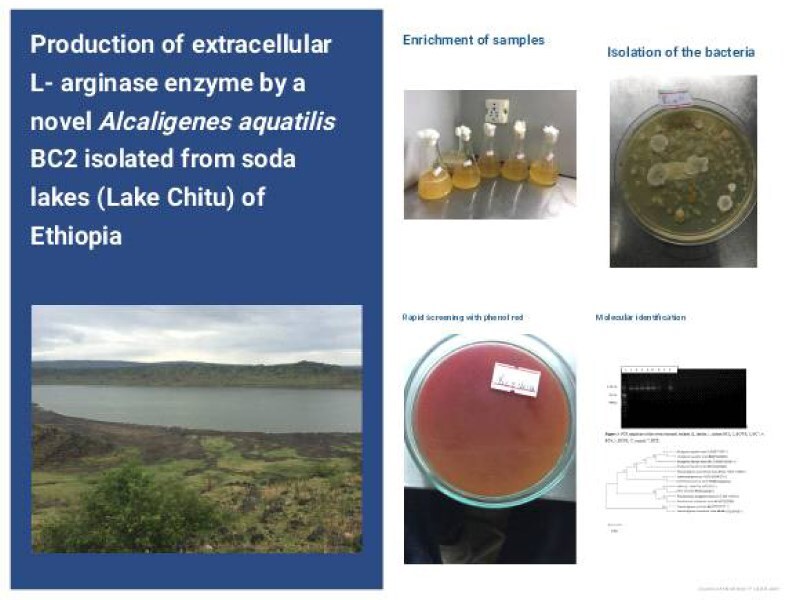

## Introduction

A manganese-containing enzyme termed L-arginase (EC 3.5.3.1), also known as L-arginine amidohydrolase, is a crucial enzyme in the urea cycle, primarily responsible for the hydrolysis of L-arginine into urea and L-ornithine. This enzymatic reaction plays a significant role in various physiological processes, including nitrogen metabolism and the regulation of nitric oxide (NO) synthesis (Clemente et al., 2020; Hassabo et al., 2022). In humans and most mammals, there are two types of arginase: arginase I (ArgI), predominantly found in the liver and involved in the fifth and final step of the urea cycle, and arginase II (ArgII), which is distributed in various tissues, including the kidneys and vascular endothelium, whose function may include NO and polyamine metabolism (Ren et al., 2022). L-Arginase is also found in different organisms, including fishes, bacteria, fungi, yeast, actinomycetes, algae, and plants (Wagemaker et al., 2005).

L-Arginine is a non-essential amino acid for humans and mice because it can be synthesized from citrulline in two steps via the urea cycle enzymes argininosuccinate synthetase 1 (ASS 1) and argininosuccinate lyase (Al-Shahari et al., 2021). Some human cancers do not express ASS and thus are unable to synthesize arginine from citrulline. Therefore, it has been suggested that an arginine-degrading enzyme may be effective in the eradication or control of arginine auxotrophic cancers (Feun et al., 2015), such as melanomas and hepatocellular carcinomas. Tumor cells require arginine for proliferation and metastasis. But in the presence of L-arginase enzyme, L-arginine is catabolized into L-ornithine and urea, inhibiting nucleic acid and protein synthesis, ultimately resulting in apoptosis of cancer cells. Normal cells can synthesize L-arginine so remain unaffected (Swanson et al., 2019).

Microbial sources of therapeutic enzymes have been preferred over plants and animals due to their cost-effectiveness, ecofriendly nature, susceptibility to genetic manipulation, and ease of process modification and purification (Zolfaghar et al., 2019). Given the scarcity of sources for therapeutic enzymes, there is an ongoing global effort to explore new organisms. This endeavor aims to identify microbial strains that can produce these therapeutic enzymes in high yields and possess unique characteristics (AS El-Sayed et al., 2014).

Extremophilic bacteria found in soda lakes create enzymes that are suited to high salinity and alkalinity. These halo-alkaliphilic enzymes are extremely stable and active in severe environments where other enzymes would normally denature. Their resilience makes them useful for therapeutic applications by improving stability during formulation, storage, and physiological function. They frequently have special catalytic qualities as well, which may lead to new therapeutic applications (DasSarma & DasSarma, 2015; Sorokin et al., 2014). Enzymes from extremophiles, including those from soda lakes, have shown antimicrobial and anticancer activities, making them candidates for developing novel therapeutics (Kulkarni et al., 2019). L-Arginase is one of them to arrest the growth of a wide range of arginine-dependent cancer cells. Therefore, the objective of this study was to screen potential L-arginase–producing bacteria from soda lake of Ethiopia.

## Materials and Methods

### Description of the Study Area

The current study was focused on the Ethiopian Rift Valley Lake Chitu, which is widely renowned for its extremophile and alkaline conditions. Lake Chitu is a tropical soda lake near Shashamane, about 287 km south of Addis Ababa, at 7°24′N 38°25′E with an elevation of 1,600 m above sea level. It is located about 1 km southwest of Lake Shala in the Ethiopian Rift Valley’s Abijata-Shala Lakes National Park (Ogato et al., 2015). Lake Chitu is rich in microbial composition (Jeilu et al., 2022a). Despite its high pH, saline-alkaline nature, and high phosphate concentration, nitrogen compounds are often limiting in the lake (Ogato et al., 2014). The lake has a stable, high pH around 10–10.5, reflecting its strongly alkaline nature due to elevated concentrations of carbonate and bicarbonate ions. Salinity levels are also high, with reported values around 3.7%–5.8%, contributing to its saline environment (Jeilu et al., 2022a; Melese & Debella, 2023).

The high concentrations of carbonate, bicarbonate, sulfate, fluoride, and other ions result from the lake’s closed basin hydrology, where evaporation exceeds inflow, leading to the accumulation of dissolved salts. These elevated ion levels create a highly alkaline and saline habitat that defines the lake as an extreme environment. Such conditions influence microbial community composition and metabolic adaptations, as only extremophiles can thrive under these physicochemical stresses (Mebrat and Etisa, 2023).

### Sample Collection

A total of 31 water samples were collected randomly from Lake Chitu, Rift Valley Lake of Ethiopia. The samples were collected from various locations across the lake to capture spatial variability in microbial populations and water chemistry. Specifically, sampling sites included both nearshore areas and points located towards the middle of the lake to represent different ecological zones. All water samples were collected at the surface layer, approximately 30 cm below the water surface, to avoid surface debris and ensure consistency across sites.

Samples at each site were collected aseptically, stored in sterilized 250 ml glass bottles, and put in an ice box at 4°C until taken to the University of Gondar microbial and cellular laboratory. The samples were kept at 4°C until processed.

### Isolation, Screening, and Identification of L-Arginase–Producing Bacteria

#### Isolation and screening of L-arginase–producing bacteria

The enrichment and isolation of L-arginase–producing bacteria were done by the protocol of Unissa et al. (2015a). For enrichment and isolation processes, 1 ml of each water sample was serially diluted 10 times using sterile saline solution and inoculated into the sterilized enrichment medium used for its isolation, having the following compositions (g/L): glucose 5.0, yeast extract 5.0, peptone 5.0, K_2_HPO_4_ 1.0, and L-arginine 10.0 in 250-ml flasks and then incubated at 30°C and 200 rpm on a rotary shaker. After sufficient mixing for 48 hr, the medium was plated in sterile Petri dishes with the above medium, including 20.0 g/l agar, and incubated at 30°C at pH 7. Fluconazole 50 µg/ml was used as an antifungal agent.

After 48 hr of incubation at 30°C, bacterial colonies with various macroscopic features were chosen from the plates based on colony size, texture, and macroscopic characteristics. Then, bacterial isolates were screened for L-arginase activity by streaking individually on mineral arginine agar media containing KCl 0.5%, MgSO_4_ 0.5%, KH_2_PO_4_ 1.0%, FeSO_4_ 0.1%, ZnSO_4_ 0.1%, and L-arginine 1.0%, along with 2% agar and phenol red reagent, incubated at 30°C and pH 7 for 12–48 hr (Unissa et al., 2015b). Arginine degradation causes a pink zone on phenol red plates primarily because the breakdown of arginine leads to the production of alkaline compounds that raise the pH of the medium. Colonies with the pink zone were considered the arginine-degrading species and were subsequently sub-cultured several times to get the pure colonies and to select for further screening activity.

### Production of Extracellular L-Arginase in Submerged Fermentation Method

For the production of extracellular enzymes by the selected bacterial isolates, 10 ml (8 × 10^8^ cells/ml) of freshly generated culture was inoculated into a 250 ml Erlenmeyer flask having the production medium (100 ml) containing glucose 1%, K_2_HPO_4_ 0.1%, L-arginine 0.5%, water 100 ml, pH 7.0, and incubated for 24 hr in an incubator shaker at 200 rpm at 30°C (Zhang et al., 2013). Following cultivation, the culture broth was centrifuged at 10,000 rpm for 30 min at 4°C. As the crude enzyme, the cell-free supernatant was used.

#### Estimation of L-arginase activity by urea assay

L-Arginase activity was investigated using colorimetric measurements of the liberated urea concentration by the diacetyl monoxime method at 520 nm wavelength (Langenfeld et al., 2021). In this test, urea reacts with diacetyl monoxime in the presence of sulfuric and phosphoric acids and ferric chloride forming a pink-colored complex. The absorbance (optical density; OD) of this complex is determined by a microprocessor UV–vis double beam spectrophotometer (Abron Instruments, India). To determine the L-arginase activity, a reaction mixture containing 0.2 ml of glycine buffer (pH 9.0), 0.5 ml of crude enzyme, and 0.1 ml of MnCl_2_ was incubated for 10 min at 37°C.

The hydrolysis reaction was accomplished by incubating activated L-arginase with 0.1 ml of L-arginine for 30 min at 37°C. After 30 min, the process was stopped by mixing in 1 ml of perchloric acid solution. Then, 1 ml of sample aliquot was added to the mixed color and mixed acid reagent, and the tubes were firmly covered and placed in a boiling water bath. After 20 min, the solution was removed, cooled to room temperature, and the OD was measured at 520 nm. Reagents without sample aliquots were used as a blank. The urea produced from the prepared urea curve was determined by varying the urea concentration from 0.1 to 0.5 mM and plotting a visual density graph against the urea concentration to extrapolate the arginase activity. Substrate without L-arginase was used as a control. One unit (U) of L-arginase activity was defined as the amount of enzyme required to release 1 μmol of urea/mL under the assay conditions.


\begin{eqnarray*}
&& {\mathrm{Enzyme\,\,{\mathrm{ activity}}}}\left( {\frac{{{\mathrm{ U}}}}{{{\mathrm{ml}}}}} \right)\\
&&\quad = \frac{{{\mathrm{\mu \,\,moles\,\,of\,\,{urea}\,\,{\mathrm{ released}}}}}}{{{\mathrm{Time\,\,of\,\,{enzyme}\,\,{axtion}}} \times {\mathrm{volume\,\,of\,\,{\mathrm{ enzyme}}}}\left( {{\mathrm{ml}}} \right)}}.
\end{eqnarray*}

The enzyme protein content was determined by the Bradford method (Kruger, 2009), with an absorbance of 595 nm, and bovine serum albumin (BSA) was used as a standard protein. The Coomassie Brilliant Blue G-250 dye binds to proteins, causing a shift in the dye’s color from brown to red. The standard curve was prepared by preparing BSA in different concentrations (0.1–0.5 mg/ml).

### Identification of L-Arginase–Producing Bacteria

#### Biochemical characterization of the isolates

The isolated bacteria were characterized by colony morphology, Gram’s reaction, catalase test, TSI fermentation tests, urease test, and several other biochemical tests (Saraf, 2017).

#### Molecular characterization of bacterial isolate

##### DNA extraction

The Qiagen bacterial DNA extraction kit (Siegen, Germany) was used to extract the genomic DNA following the manufacturer’s protocol. The concentration and quality of the extracted DNA were determined by subjecting the samples to the Thermo Scientific NanoDrop 2000 spectrophotometer (Thermo Scientific, USA) and gel electrophoresis. Three microliters of each sample were stained using the SYBR green dye with ethidium bromide and loaded in a 2% agarose gel at 80 V in 0.5X TAE buffer (Bio Lab) for 30 min (Kyule et al., 2022). Distilled H_2_O was used as a control in the gel run. The isolated DNA samples were stored at −20°C for PCR amplification.

##### PCR amplification

The isolated DNA was amplified based on a protocol developed by Zolfaghar et al. (2019). The 16S rRNA gene was amplified by thermal cycler (MasterCycler Gradient, Eppendorf) using the universal primers 27F (5′-AGA GTT TGATCCTGG CTC AG-3′) and 1492R (5′-CAC GGA TCC TAC GGG TAC CTT GTT ACG ACT T-3′).

For the PCR reaction, 25 μl reaction mixtures, 12.5 μl 2X Taq™ master mix (Promega), 0.5 µl of 1 µM of each forward and reverse primer, 10.5 μl of double distilled water, and 1 µl of genomic DNA template (200 ng) were used for amplification. The amplification condition was initial denaturation at 95°C for 5 min, followed by 30 cycles of final denaturation at 94°C for 60 s, annealing at 57°C for 60 s, and extension at 72°C for 60 s, with a final 7-min extension at 72°C. The final PCR products were visualized on 1% agarose gel electrophoresis to confirm whether the PCR amplification was successful or not.

##### Sequence and phylogenetic analysis

The PCR amplicons were purified and sequenced at Macrogen Europe BV, Netherlands, using the Sanger sequencing method. The sequenced raw DNA chromatogram sequences were examined by using the BioEdit software (Tippmann, 2004) and stored in FASTA format. After removing poor-quality sequences from the 3′ and 5′ sequence ends, the forward and reverse sequencing products were assembled.

The resulting 16S rRNA sequence was aligned with reference sequences retrieved from the National Center for Biotechnology Information (NCBI) database using the Basic Local Alignment Search Tool (BLAST). MEGA 11 software was used for phylogenetic analysis of the sequence. Kimura’s two-parameter model was used to compute pairwise evolutionary distances between them. The neighbor-joining method was used to generate a phylogenetic tree from distance matrices, and the topology was verified using a CLUSTAL W bootstrap analysis with 1,000 replicates (Saitou & Nei, 1987). The obtained sequence was submitted with unique codes for deposition in GenBank.

### Optimization of the L-Arginase Production Medium by *Alcaligenes aquatilis* BC2

To increase *Alcaligenes aquatilis* BC2’s production of L-arginase, several factors were tested. Just a single parameter is optimized at a time, while all other parameters stay unchanged. Once one aspect was optimized, it was utilized in the subsequent optimization process at its best level. The extracted enzyme was assayed for urea using the diacetyl monoxime method (Langenfeld et al., 2021).

#### Optimization of nutritional factors

##### Carbon sources

Carbon sources such as glucose, galactose, sucrose, maltose, and lactose were added to the fermentation medium at a concentration of 1% (w/v). Fermentation medium without carbon source was used as a control. The flask was inoculated with 1% inoculum and incubated for 24 hr at 37°C in an incubator shaker at 120 rpm. The best carbon source was further optimized by changing the concentration to 1, 2, 3, 4, and 5% w/v.

##### Nitrogen sources

As nitrogen sources, 1% w/v of peptone, yeast extract, tryptone, sodium nitrate, and ammonium chloride were employed. Fermentation medium without nitrogen source was used as a control. In addition, the amount of the selected nitrogen source was optimized using 1, 2, 3, 4, and 5% w/v.

##### Effect of arginine concentration

A different L-arginine concentration (0.5, 1, 1.5, 2, 2.5, and 3% w/v) was used for the production of the L-arginase enzyme.

#### Optimization of other parameters for L-arginase production

##### Effect of pH

The effect of initial pH on enzyme production was determined by adjusting the fermentation medium to various pH values (5, 6, 7, 8, 9, and 10).

##### Effect of temperature

The effect of temperature on arginase production was studied by varying it from 25°C to 50°C with an interval of 5°C while keeping other factors constant.

##### The effect of incubation period

The effect of the incubation period on arginase production was optimized using 24, 48, 72, 96, and 120 hr.

##### The effect of agitation period

The culture was treated to different agitations of 100, 150, and 200 rpm to ensure homogeneous circulation of nutrients, and its effect on enzyme synthesis was measured.

##### The effect of inoculum size on arginase production

Different inoculum sizes (1–5% w/v) were provided for the media in the production of enzymes.

### Data Analysis

Microsoft Excel 2013 was used for generating standard curves. GraphPad Prism software 10.4.2 was used for plotting the graphs. The Statistical Package for Social Science version 25 software was used for the descriptive analysis in mean ± SD of the triplet data. Comparison of the mean and significant difference in the mean values was analyzed and compared by one-way ANOVA followed by Tukey’s post hoc analysis. Statistical differences at *p* < .05 were considered statistically significant.

## Result and Discussion

### Isolation and Screening of L-Arginase–Producing Bacteria

Extreme environments such as soda lakes are home to unique microorganisms that play an essential role in the production of potentially therapeutic enzymes (Mesbah, 2022). According to Jeilu et al. (2022b), the distinctive microbial diversity in Ethiopian soda lakes has resulted in the discovery of new enzymes that are stable and active in alkaline environments, including amylase and xylanase.

Samples from Soda Lake were subjected to enrichment and the streak plate methods. Based on colony morphology, 102 pure bacterial colonies (isolates) were isolated from 31 water samples from different sites in Lake Chitu after repetitive streaking of each colony (Parumasivam et al., 2024). All isolates were tested for primary screening of L-arginase production. Mineral arginine agar medium supplemented with phenol red was used for qualitative screening.

**Fig. 1:**
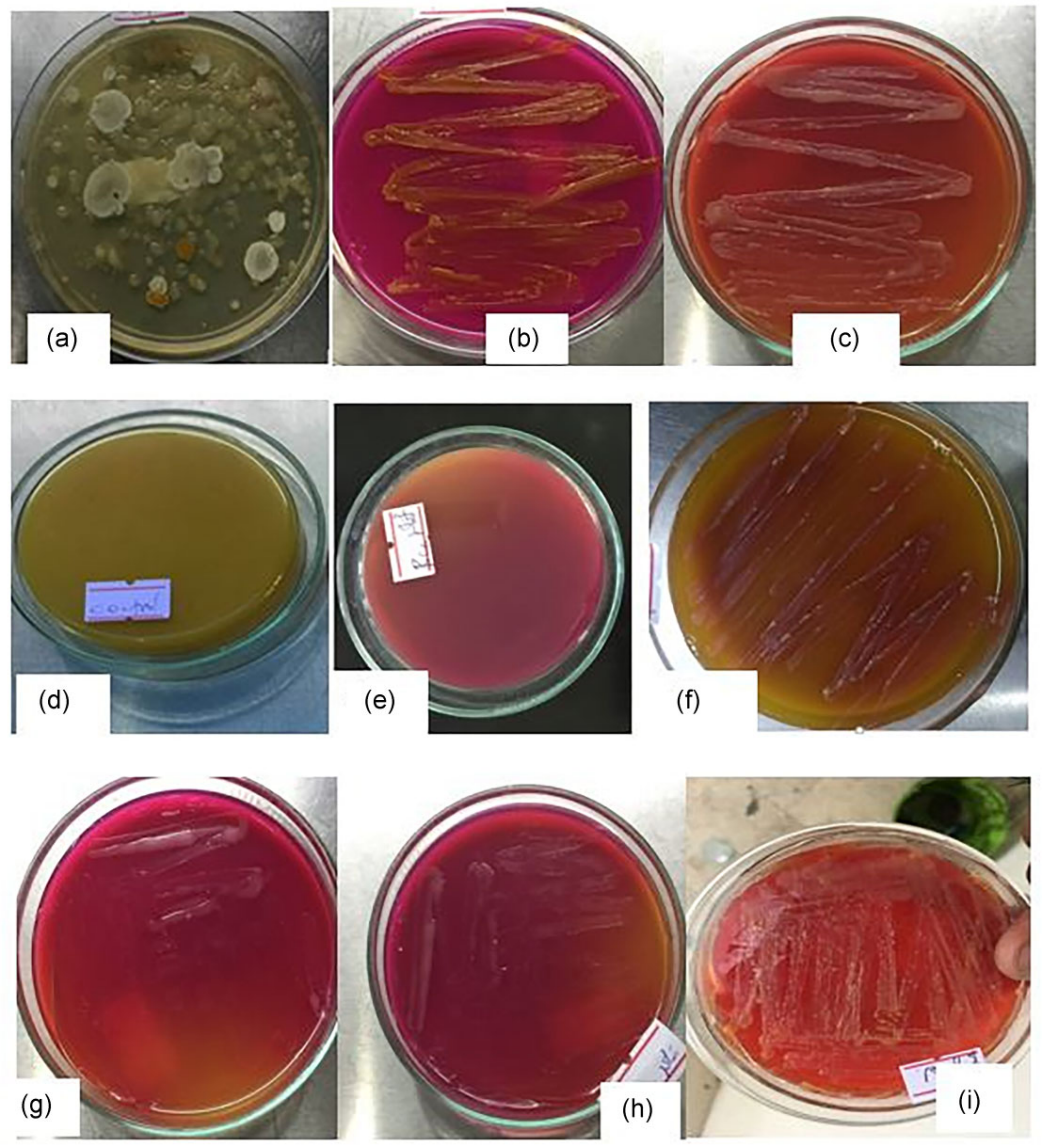
Isolation and screening of L-arginase–producing bacteria. (a) Different colonies on primary screening plate; (b) positive isolate for L-arginase production (isolate BC2); (c) positive isolate for L-arginase production (BC7); (d) control-uninoculated medium showing the original color in the absence of bacterial growth; (e) positive isolate for L-arginase production (isolate BC4); (f) positive isolate for L-arginase production (isolate BCG8); (g) positive isolate for L-arginase (isolate BC3); (h) positive isolate for L-arginase (isolate BCW8); and (i) positive isolate for L-arginase (BT2).

During the primary screening, 18 isolates that changed the medium color from yellow to pink within 12–48 hr of incubation were identified as potential L-arginase–producing bacteria (Fig. 1). Phenol red has been widely used in previous studies for the rapid isolation and screening of L-arginase producers (Agbaje et al., 2022; Alzahrani, 2020; Kassab, 2024b; Selim et al., 2024). In this study, the pink coloration of the medium indicated that these isolates hydrolyzed L-arginine by producing the L-arginase enzyme. This color change results from the hydrolysis of L-arginine into ornithine and urea, which raises the pH of the medium. According to Zhang et al. (2013), formation of pink color around the colonies is due to the production of L-arginase.

Among those 18 L-arginase–producing bacteria, seven isolates that exhibited pink coloration within 24 hr were classified as showing a “rapid” color change and selected for further quantitative analysis. To ensure accuracy and avoid false positives caused by protein hydrolysis or arginine deiminase (ADI) activity, all isolates were subjected to secondary quantitative screening.

#### Biochemical and morphological characterization

The seven isolates were grown in nutrient agar to study morphological features of the isolates. All the isolates have circular shapes. The Gram staining result showed that all isolates were Gram negative. Different colony morphology was observed among the seven isolates (Table 1).

**Table 1.** Morphological Characteristics of L-Arginase–Producing Bacterial Isolates

Isolate code	Shape	Color	Texture	Elevation	Size	Margin
BC2	Circular	Grayish	Moist	Raised	Medium	Wavy
BC3	Circular	Yellow	Shiny	Slight raised	Small	Entire
BC4	circular	White	Smooth	Flat	Medium	Entire
BC7	Circular	White	Moist	Raised	Medium	Irregular
BCW8	Circular	White creamy	Moist	Raised	Small	Entire
BCG8	Circular	Brown	Smooth	Raised	Medium	Entire
BT2	Circular	White	Mucoid	Raised	Medium	Entire

Based on their biochemical characterization, all the isolates were positive for catalase and citrate utilization test. Among the isolates, only BC2, BT2, and BC4 were positive for urease test (Table 2).

**Table 2.** Biochemical Characterization of Isolates

Isolates	G-staining	Biochemical tests						
		Urease	Citrate	Catalase	TSI			
					Butt	Slant	H_2_S prod	Gas prod
BC2	−ve	+ve	+ve	+ve	R	R	−ve	−ve
BC7	−ve	−ve	+ve	+ve	R	R	−ve	−ve
BC3	−ve	−ve	+ve	+ve	Y	R	−ve	−ve
BC8W	−ve	−ve	+ve	+ve	Y	Y	+ve	−ve
BC8G	−ve	−ve	+ve	+ve	R	R	+ve	−ve
BC4	−ve	+ve	+ve	+ve	Y	R	−ve	+ve
BT2	−ve	+ve	+ve	+ve	Y	R	+ve	+ve

**Key: G-staining**: Gram staining result (−ve = Gram-negative, +ve = Gram-positive).

Urease, citrate, and catalase indicate the presence (+ve) or absence (−ve) of enzyme activity. **Triple sugar iron (TSI) test: Butt**: the anaerobic (bottom) portion of the TSI agar tube; color indicates sugar fermentation (R = red, alkaline; Y = yellow, acid). **Slant**: the aerobic (slanted) surface of the TSI agar tube; color indicates sugar fermentation (R = red, alkaline; Y = yellow, acid). **H₂S prod**: hydrogen sulfide production (+ve = positive, −ve = negative). **Gas prod**: gas production (+ve = positive, −ve = negative).

For the triple sugar iron (TSI) test, isolates BC3, BCW8, BC4, and BT2 have changed the color of butt to yellow, which indicates that those isolates do ferment glucose. The color change formed in the butt is due to the subsequent reduction in pH by the formation of acid (Islam & Khanam, 2021). Among the seven isolates, only isolate BCW8 shows yellow (positive) in the slant, which indicates the isolate ferments not only glucose but also lactose and sucrose.

#### L-Arginase activity of isolates

The seven isolates were subjected to submerged fermentation at shake flask level for quantitative assay of L-arginase production colorimetrically using a spectrophotometer at 520 nm. The activity of L-arginase was quantified by the amount of urea released from the L-arginine hydrolysis reaction by the enzyme. The reaction aliquots were analyzed by the diacetyl monoxime method to measure the amount of urea liberated from the degradation of L-arginine (Kassab, 2024a). In this study, isolate BC2 demonstrated the maximum mean value of L-arginase activity **(**92.45 ± 0.19 U/ml), followed by isolate BCW8 (59.29 ± 0.66 U/ml), as presented in Table 3. One-way ANOVA results indicate that there is a significant difference between the seven isolates (*p*-value < .05) for L-arginase activity. There was no significant difference between isolates BC4 and BCG8 in their soluble protein content. This study demonstrates significantly higher L-arginase activity compared to previous work with marine isolates (Selim et al., 2024) and soil samples (Nadaf et al., 2019). The highest protein concentrations were recorded in isolate BC7 (47.96 ± 0.45 µg/ml), followed by BCW8 (40.36 ± 0.01 µg/ml). While the enzymes produced by isolates BC3 and BCG8 (7.07 ± 0.87 and 12.80 ± 1.01), respectively, were recorded as significantly lower L-arginase activity. It has been observed that water samples from soda lakes in the rift valley of Ethiopia contain novel L-arginase–producing isolates.

**Table 3.** L-Arginase Activity and Protein Determination of Isolates

Isolate code	L-arginase activity (U/ml)	Protein concentration (µg/ml)
BT2	37.59 ± 1.25^d^	31.90 ± 0.003^f^
BC3	7.07 ± 0.87^g^	35.36 ± 0.006^d^
BC7	44.64 ± 0.83^c^	47.96 ± 0.451^a^
BC8G	12.80 ± 1.01^f^	33.35 ± .005^e^
BCW8	59.29 ± 0.66^b^	40.36 ± 0.01^b^
BC4	29.22 ± 0.87^e^	33.63 ± 0.014^e^
BC2	92.46 ± 0.19^a^	39.5 ± 0.1^c^

*Note*. Within a column, the mean difference values with different superscripts are significantly different at *p* ≤ .05 (Tukey HSD analysis).

#### Molecular identification of L-arginase–producing bacteria

Significant morphological variation among microorganisms makes it difficult to identify bacteria based solely on their morphology. Consequently, this study employed molecular identification to identify specific organisms. The conserved genomic region of 16S rRNA, found in the smallest subunit of ribosomal RNA (30S), was utilized to classify and identify bacteria (González et al., 2024; Paul, 2023).

**Fig. 2:**
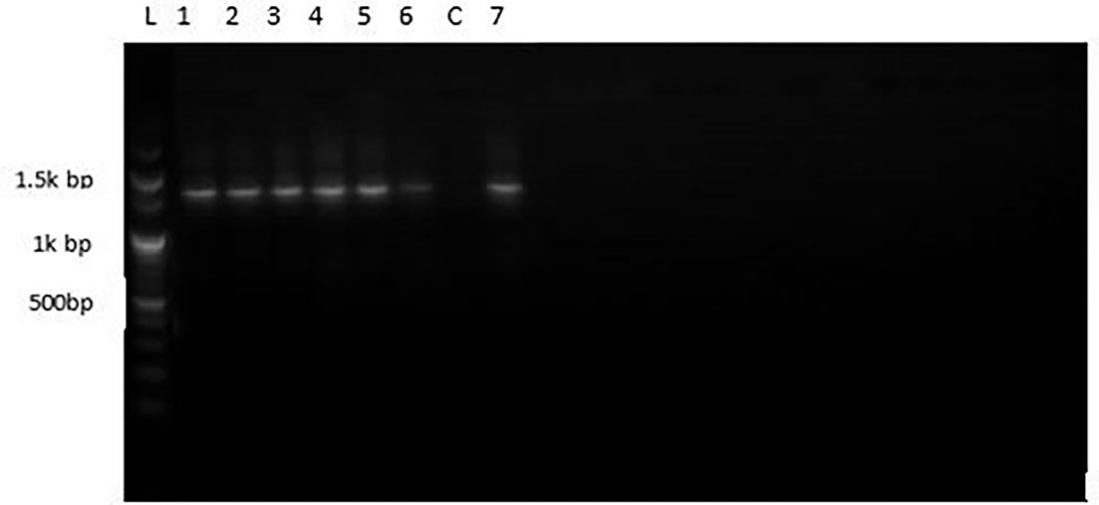
PCR amplicon of the seven bacterial isolates (L, ladder; lane 1, isolate BC2; lane 2, BCW8; lane 3, BC7; lane 4, BC4; lane 5, BCG8; lane 6, BC3; lane C, control and lane7, BT2).

In this study, genomic DNA from the selected bacterial isolates was extracted and amplified using PCR with two universal primers. The amplified DNA samples from the seven isolates were run on a 1% agarose gel electrophoresis, with all samples showing a band at approximately 1,400 bp (Fig. 2). The base pair size of the PCR products from our isolates was consistent with results reported by Unissa et al. (2015a), where 1.4 kb of DNA from ADI-producing isolates were screened. The nucleotide sequence amplification of the 16S ribosomal RNA typically yields a sequence of about 1,500 bp (Church et al., 2020). This indicates that the PCR amplicons from the present study are of good quality and provide valuable taxonomic information.

The PCR products of the 16S rRNA gene from the seven isolates were subsequently subjected to sequencing. The BLAST analysis results obtained from the NCBI database using BLASTN revealed that isolate BC2 shares 97.30% sequence identity with *A. aquatilis* strain 2JA, suggesting its classification within the *A. aquatilis* species or a closely related species, isolate BCW8 showed 97.97% similarity with *Paenalcaligenes suwonensis* strain L5 (PP843540.1), and the L-arginase–producing isolate BC7 exhibited 99.92% homology with *Alcaligenes faecalis* strain B16. Isolate BC3 had 82.44% homology with *Paenalcaligenes* sp. strain UN24, as shown in Table 4. This lower similarity may be attributed to the strain being novel or to the poor quality of the DNA sequence. Other strains of *Alcaligenes* isolated from the rhizosphere have also been documented among L-arginase–producing bacteria (Agbaje et al., 2022).

**Table 4.** The BLAST Results of Our Isolates

Isolate	Accession number	Query	*E*-value	% identity	Similar with
BC2	PQ 184653.1	100%	0.00	97.30	*Alcaligenes aquatilis* strain 2JA **(**MZ713000.1**)**
BCW8	PQ 187436.1	100%	0.00	97.97	*Paenalcaligenes suwonensis* strain L5 (PP843540.1)
BC3	PQ 187437.1	97%	6e-120	82.44	*Paenalcaligenes* sp. strain UN24 (KP277115.1)
BT2	PQ 203971.1	94%	0.00	100	*Vibrio sp*. Strain P26 (KR075025.1)
BC7	PQ 222569.1	99%	0.00	99.92	*Alcaligenes faecalis* strain B16 (MG234448.1)
BCG8	PQ 222574.1	100	0.00	100	*Janthinobacterium* sp. HHS32 (AJ846273.1)
BC4	PQ 222 589	100	0.00	98.04	*Pseudomonas alcaligenes* strain ATCC (NR114472.1)

The phylogenetic tree was constructed by MEGA 13 software using the sequences of our isolate and other related sequences by the neighborhood-joining method. The phylogenetic analyses were done by maximum likelihood and the Kimura 2-parameter model with 1,000 replication bootstraps to estimate the tree topology (Challa & Neelapu, 2019).

**Fig. 3:**
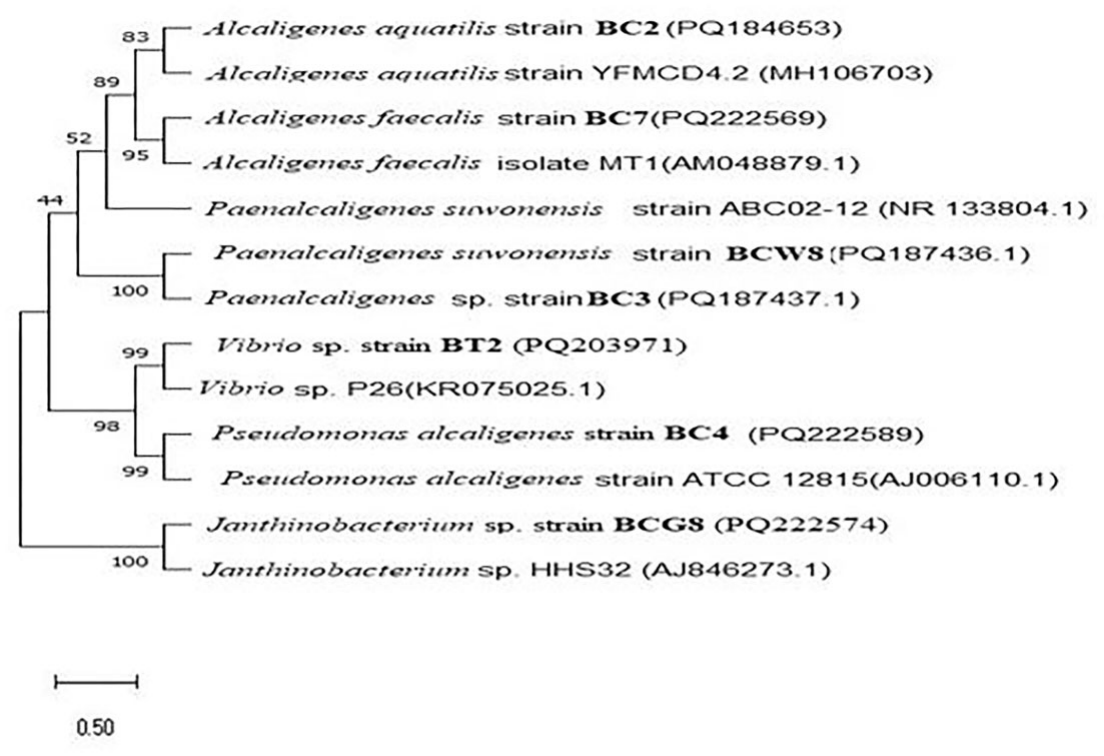
The phylogenetic tree of isolates was deduced through the neighbor-joining method, with evolutionary distances calculated based on the Kimura 2-parameter model. A consensus tree derived from 1,000 bootstrap replicates was used to illustrate the evolutionary relationships among the analyzed taxa. The isolates examined in this study are highlighted in bold.

Isolate BC2 and strain 2JA of *A. aquatilis* form a distinct clade on the phylogenetic tree (Fig. 3) due to their close relationship, suggesting they share a recent common ancestor and likely possess similar genetic and phenotypic characteristics. *Alcaligenes aquatilis* is a rod-shaped, Gram-negative bacterium primarily found in water. The work of Cao et al. (2022), who isolated *A. aquatilis* from water, reveals our findings.

*Alcaligenes faecalis* strain IAM 12369 and isolate BC7 comprise a closely associated clade, indicating an evolutionary link, although they are more distantly related to strains of *A. aquatilis*. The current study indicates that *Alcaligenes* species are capable of producing L-arginase. Similar results were observed in Agbaje et al. (2022), who used the *Alcaligenes faecalis* species to produce L-arginase, and *Vibrio* species were also identified as L-arginine-hydrolyzing isolates, consistent with the findings of Unissa et al. (2015a), who screened *Vibrio alginolyticus* from marine sediments.

Our investigation revealed that isolate BC2, *A. aquatilis* BC2, is the most effective strain of bacteria for L-arginase production compared to other isolates. Notably, it also produces L-arginase, a capability that has not been previously investigated; thus, we have chosen it for further media optimization.

### Optimization of the Production Medium

#### Optimization of nutritional factors for L-arginase production by *A. aquatilis* BC2

##### Carbon sources

**Fig. 4:**
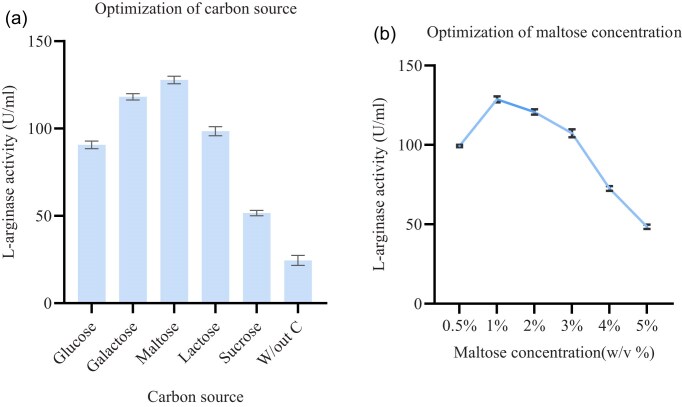
The effect of different carbon sources (a) and the effect of different maltose concentrations (b) on L-arginase activity. Data are presented as mean ± standard deviation (SD) of three independent experiments (*n* = 3).

Enzyme production and bacterial growth are significantly impacted by the kind and quantity of carbon source used. The carbon source in this investigation was a 1% (w/v) addition of glucose, galactose, sucrose, maltose, and lactose to the fermentation medium, and the control was fermentation medium devoid of a carbon source. Maximum enzyme production was observed (127.52 U/ml), when maltose was introduced to the fermentation medium. According to recent studies by Nadaf and Vedamurthy (2020) and Selim et al. (2024), who worked with *Bacillus licheniformis* OF2 and *Pseudomonas* sp. strain PV1, respectively, the addition of maltose as a carbon source maximizes the synthesis of L-arginase, and our finding is similar to this report. However, Gautam et al. (2022), working with *Lactobacillus acidophilus*, found that lactose was the best carbon source, while sucrose also supported the highest synthesis of L-arginase (Agbaje et al., 2022). As shown in Fig. 4a, the least effective carbon source was sucrose (51.49 U/ml). Based on our findings, varying the concentration of maltose has a profound effect on L-arginase production. The maximum production of L-arginase was recorded at 1% maltose (128.74 U/ml) and further increase in maltose concentration leads to the decrease in L-arginase production (Fig. 4b). This reduction may be caused due to catabolite repression of glucose. When microorganisms are exposed to excessive maltose, the degradation of maltose results a sharp rise in glucose levels, which can further prevent the transport and utilization of maltose (Jansen et al., 2004).

##### Nitrogen sources

**Fig. 5.**
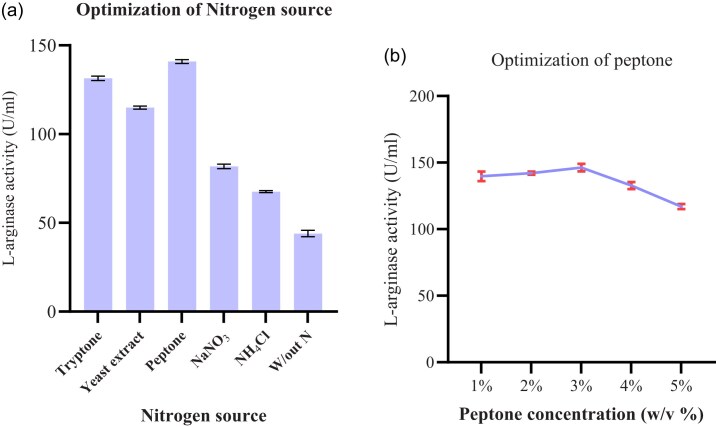
The effect of different nitrogen sources (a) and the effect of different peptone concentrations (b) on L-arginase activity. Data are presented as mean ± standard deviation (SD) from three independent experiments (*n* = 3).

The addition of other nitrogen sources with L-arginine to the fermentation medium showed a significant effect on the enzyme production. As nitrogen sources, 1% w/v of peptone, yeast extract, tryptone, sodium nitrate, and ammonium chloride were employed. Fermentation medium without nitrogen source was used as a control. As presented in Fig. 5a, peptone supported the maximum enzyme production (140.9 U/ml) followed by tryptone and yeast extract. The lowest enzyme production (67.59 U/ml) was recorded when ammonium chloride was used as a carbon source. The findings of this study indicated that the inorganic nitrogen sources revealed the lowest L-arginase production than those organic sources. This might be due to the abundant nature of organic sources in different nutrients, which are used for the growth of microorganisms and easy to be hydrolyzed by microbes. In this study, use of peptone showed the highest L-arginase activity.

Our result is in line with the study of Selim et al. (2024), who use peptone as a nitrogen source for the production of the highest L-arginase from marine *B. licheniformis* OF2. On the contrary, yeast extract was found to be the best nitrogen source as reported by Agbaje et al. (2022), Gautam et al. (2022), and Nadaf and Vedamurthy (2020) in their findings. A report from other scholars indicated that the use of soybean meal as a nitrogen source maximized the synthesis of enzymes (Unissa et al., 2015c).

The optimal concentration of peptone as a nitrogen source for the L-arginase activity (Fig. 5b) was shown at 3%.

##### Effect of arginine concentration

**Fig. 6:**
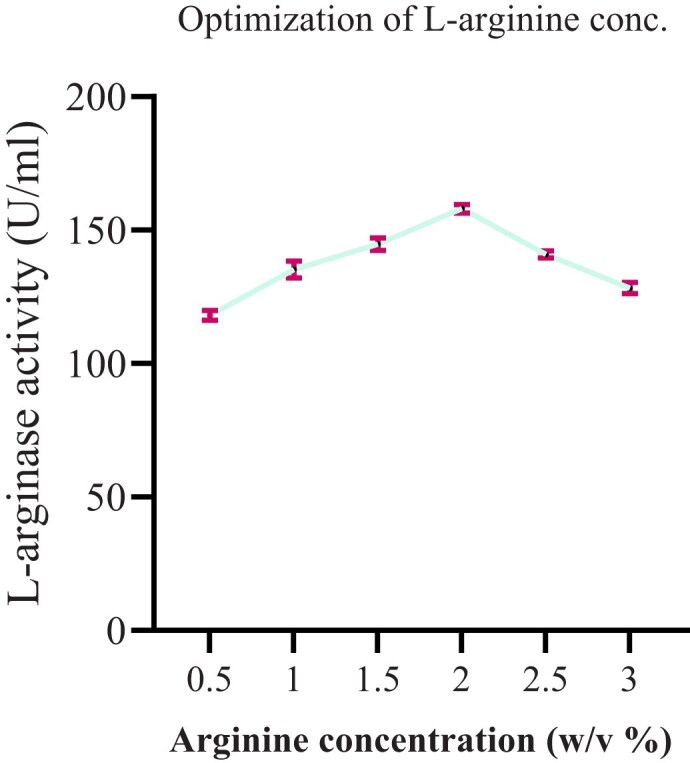
The effect of L-arginine concentration on L-arginase activity. Data are presented as mean ± standard deviation (SD) from three independent experiments (*n* = 3).

A different L-arginine concentration (0.5, 1, 1.5, 2, 2.5, and 3% w/v) was used to produce the L-arginase enzyme. The maximal L-arginase activity (158.01 U/ml) was observed at 2% L-arginine concentration. Equivalent results were recorded in the study of Unissa et al. (2018) using *Idiomarina sediminium* H1695. The lowest L-arginase activity (Fig. 6) was showed at the initial concentration 0.5%. Further increase of substrate from the optimum leads to the reduction of L-arginase activity in this study. This may be due to the inhibitory effect of L-arginine for the production of enzymes. In contrast, the study conducted by Agbaje et al. (2022) uses 6% L-arginine concentration for optimal activity of L-arginase.

#### Optimization of other parameters for L-arginase production

##### Effect of pH

**Fig. 7:**
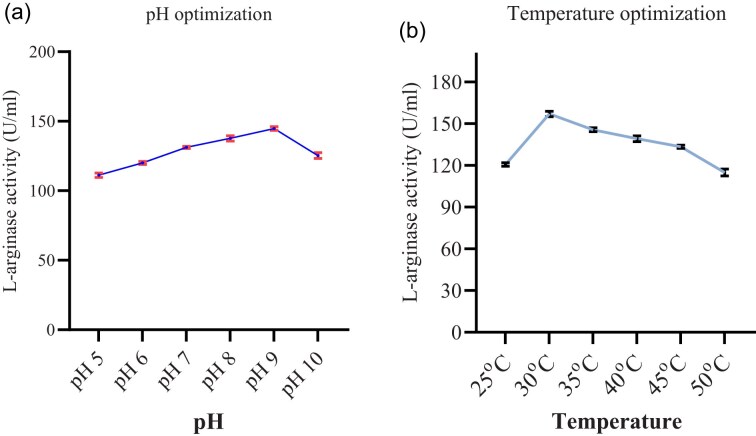
The effect of pH on L-arginase activity (a) and the effect of T^o^ on L-arginase activity (b). Data are presented as mean ± standard deviation (SD) of three independent experiments (*n* = 3).

The initial pH of the fermentation media speeds up the growth and production of enzyme by reducing the duration of lag phase. To determine the effect of initial pH, the production medium was adjusted to various levels of pH (5, 6, 7, 8, 9, and 10). In this study, we observed that the production of L-arginase substantially increased from pH 5 to pH 9. The highest L-arginase activity (144.75 U/ml) was observed at pH 9. The pH 5 gave low L-arginase production as shown in Fig. 7a. These results indicate that the bacteria isolated from Soda Lake were highly alkalophilic in nature. The optimum pH observed in this study was similar to the study of Unissa et al. (2018). Other studies also found L-arginase production at alkaline pH (Abd et al., 2024; Nadaf & Vedamurthy, 2020; Selim et al., 2024). On the contrary, Gautam et al. (2022) reported the production of L-arginase by *L. acidophilus* at pH 6.

##### Effect of temperature

The effect of temperature on the production of arginase was studied by changing the temperature from 25°C to 50°C with an interval of 5°C while keeping other factors constant. The maximum L-arginase production (157.07 U/ml) was obtained at temperature of 30°C. When the temperature decreases from the optimum, the L-arginase production decreases (Fig. 7b). This may be because variation in temperature below or above the optimum causes the microorganisms to slow down their metabolic activities due to denaturation of proteins, which leads to less enzyme production (Kabir & Ju, 2023). The results of the current study suggest that the maximum enzyme activity was recorded at 30°C. The findings of an earlier report had also indicated that L-arginase may be produced at temperatures between 27°C and 37°C (Agbaje et al., 2022). Although the ideal temperature for L-arginase production was 30°C, *A. aquatilis* may thrive and grow in a variety of temperatures, even at 60°C, and still generate an enzyme (Javia et al., 2023).

##### The effect of incubation period

**Fig. 8:**
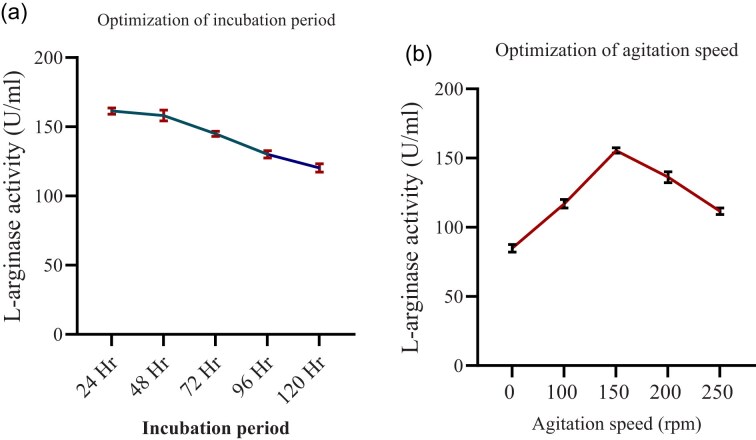
The effect of incubation period (a) and the effect of agitation speed (b) on L-arginase activity. Data are presented as mean ± standard deviation (SD) from three independent experiments (*n* = 3).

Incubation period of the fermentation process is one of the crucial factors for better enzyme production. The effect of the incubation period on arginase production was optimized using 24, 48, 72, 96, and 120 hr. The maximum production (161.4 U/ml) of L-arginase was observed at 24-hr incubation period (Fig. 8a). The L-arginase production decreased as the fermentation time exceeded. This may be attributed not only to the exhaustion of nutrients in the fermentation medium but also to possible degradation or inactivation of the extracellular enzyme over time, changes in pH affecting enzyme stability, or shifts in cellular metabolism as cells enter the stationary phase.

In this study, the maximum amount of L-arginase was generated after 24 hr of incubation, and this is in line with the findings reported previously by Nadaf and Vedamurthy (2020) as well as Gautam et al. (2022). However, our finding was different compared with the work reported elsewhere (Abd et al., 2024; Unnisa et al., 2018), as they employ different incubation times to get the highest result.

##### The effect of agitation period

The culture was treated to different agitation of 100, 150, and 200 rpm in order to ensure homogeneous circulation of nutrients. By accelerating the rate of oxygen transfer and boosting the dispersion of nutrients, agitation is a crucial component that promotes bacterial growth and the production of enzymes by allowing them to come into touch with the microbial cell (Dutta et al., 2024). In this study, the highest L-arginase production (155.47 U/ml) was recorded at 150 rpm agitation speed (Fig. 8b). This result is consistent with other reports (Agbaje et al., 2022; Nadaf & Vedamurthy, 2020). However, the L-arginase production decreases above the optimum agitation speed. This might be the result of excessive agitation speed, which damages the integrity of the cells by causing shearing stress. Turbulence caused by severe agitation might interfere with ideal diffusion patterns. This may result in confined regions with low concentrations of substrate (Leite et al., 2023).

### The Effect of Inoculum Size on Arginase Production

**Fig. 9:**
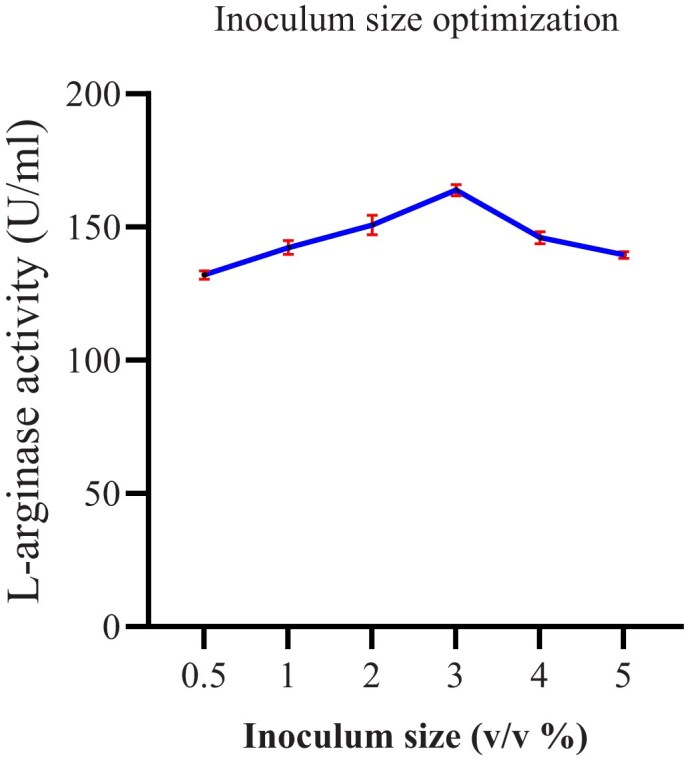
The effect of inoculum size on L-arginase production. Data are presented as mean ± standard deviation (SD) from three independent experiments (*n* = 3).

The initial inoculum size determines growth and production of enzymes by microorganisms. In this study, different inoculum sizes (1–5% w/v) were provided for the fermentation media in the production of L-arginase enzyme. The result (Fig. 9) of our study indicates that optimal L-arginase production (163.85 U/ml) was observed at 3% inoculum size. Overextending the inoculum beyond its optimum size can result in nutritional exhaustion, which lowers enzyme output. In contrast to the findings of Unissa et al. (2018), who found that 10% inoculum volume was optimal for efficient L-arginase production, the current investigation used less inoculum size.

## Conclusion

Screening and characterizing L-arginase–producing bacteria from soda lakes was the goal of the current investigation. Eighteen isolates out of the total turn the mineral arginine agar medium to pink. They are thought to be the best isolates that produce L-arginase, and seven of them quickly convert the medium. Subsequent screening of the bacterial isolates based on their enzyme activity revealed notable variations in their production of enzymes. Among the isolates, isolate BC2 has the greatest enzyme activity (92.458 ± 0.191 U/ml) and is thought to be a new L-arginase–producing bacterium. Using 16S rRNA sequencing, the isolate was further identified as *A. aquatilis* BC2. Following the optimization of several parameters, the bacteria produced a maximum amount of L-arginase (163.85 U/ml) at 1% maltose, 3% peptone, pH 9, 30°C, 24-hr incubation, 150 rpm, and 3% inoculum volume. This study suggests that soda lakes embrace new bacteria producing powerful therapeutic enzymes that can possibly be employed to assess the anticancer activity on various cancer cells for the future.

## Data Availability

The manuscript and all required data are uploaded to the web system online. If additional information is needed, the corresponding author will provide it upon request.
